# Indigenous Social Enterprises and Health and Wellbeing: A Scoping Review and Conceptual Framework

**DOI:** 10.3390/ijerph192114478

**Published:** 2022-11-04

**Authors:** Sara Hudson, Dennis Foley, Margaret Cargo

**Affiliations:** 1 Health Research Institute, University of Canberra, 11 Kirinari St., Canberra, ACT 2617, Australia; 2 School of Management, Faculty of Business, Government and Law, University of Canberra, 11 Kirinari St., Canberra, ACT 2617, Australia

**Keywords:** Indigenous social enterprise, Indigenous research methodologies, health and wellbeing, self-determination, cultural values, cultural and social determinants of health, hybridity

## Abstract

Indigenous people and communities are establishing social enterprises to address social disadvantage and overcome health inequities in their communities. This review sought to characterize the spectrum of Indigenous social enterprises in Australia, New Zealand, Canada, and the United States to identify the operational models and cultural values that underpin them and their impact on Indigenous health and wellbeing. The scoping review followed Arksey and O’Malley’s six-stage methodological framework with recommended enhancements by Levac et al. underpinned by Indigenous Standpoint Theory, and an Indigenous advisory group to provide cultural oversight and direction. Of the 589 documents screened 115 documents were included in the review. A conceptual framework of seven different operational models of Indigenous social enterprises was developed based on differing levels of Indigenous ownership, control, and management: (1) individual, (2) collective, (3) delegative, (4) developmental, (5) supportive, (6) prescriptive and (7) paternalistic. Models with 100% Indigenous ownership and control were more likely to contribute to improved health and wellbeing by increasing self-determination and strengthening culture and promoting healing than others. Indigenous social enterprises could offer a more holistic and sustainable approach to health equity and health promotion than the siloed, programmatic model common in public health policy.

## 1. Introduction

Overall, Indigenous people throughout the world experience poorer health outcomes than non-Indigenous people [1]. In attempting to address this disparity, the link between self-determination, economic development and health and wellbeing, has become increasingly recognised. In 2007, the United Nations General Assembly adopted the United Nations Declaration on the Rights of Indigenous Peoples. Article 3 of the Declaration enshrines Indigenous peoples’ right to “self-determination…and to freely pursue their economic, social and cultural development.” [2] (p. 4). The important role of self-determination in improving health and well-being is documented in the Organisation for Economic Co-operation and Development’s (OECD) wellbeing framework [3]. The framework defines economic development as a process that expands people’s choices and opportunities to live lives that they value [3]. The relationship between economic activities and wellbeing is also highlighted in the Australian Bureau of Statistics Framework for Measuring Wellbeing: Aboriginal and Torres Strait Islander Peoples, which includes nine domains encompassing, culture, heritage, family and community, education, income, economic resources and health among others [4].

In Australia, the National Aboriginal Community Controlled Health Organization (NACCHO) defines Aboriginal health as: “not just the physical well-being of an individual but…the social, emotional and cultural well-being of the whole Community…” [5] (para. 2). This understanding of health encompasses social and cultural factors, such as the quality of people’s relationships and the strength of their cultural connections and is more holistic than Western notions of health. Research by Indigenous scholars on health and wellbeing emphasize the importance of the collective and link Indigenous concepts of health and wellbeing with kinship relations, connection to Country, self-determination and cultural and spiritual practices [6,7,8]. However, holistic notions of health and wellbeing are difficult to apply in the Indigenous health space, which is characterised by top-down government-controlled programs, funding siloes and short-term contracts that do not empower Indigenous communities or meet their needs [9].

One of the ways in which Indigenous people are seeking to alleviate poverty and disadvantage and improve health and wellbeing is through social enterprises that provide employment and other opportunities for their communities [10,11,12]. Although there are different definitions of social enterprise, a common definition used in Australia, is: “organisations that are led by an economic, social, cultural, or environmental mission consistent with a public or community benefit; [that] trade to fulfil their mission; derive a substantial portion of their income from trade; and reinvest the majority of their profit/surplus in the fulfilment of their mission.” [13] (p. 347).

Within the literature there was no agreed definition of Indigenous social enterprise, though there was general consensus that Indigenous social enterprises had unique characteristics that distinguished them from other types of social enterprises. For example, Henriques et al. [14] argue that Indigenous social enterprises: “draw on community resources to maximise blended value creation to address a community’s economic, social (spiritual and social justice) and environmental issues and challenges produced by a legacy of racism and colonization.” (p. 314). Many Indigenous social enterprises are collectively owned and focus on achieving outcomes that benefit community [14]. The social enterprise model and its emphasis on using profits to support social and cultural outcomes is a type of hybrid business that is closely aligned with Indigenous cultural values of reciprocity and community mindedness [11,14,15,16]. Many authors consider Indigenous social enterprises to be a form of hybrid business, not only because they combine business and social objectives but because they combine traditional cultural values and practices with Western business methods [15,17,18]. Although social enterprise is a relatively ‘new concept’, some argue it is an ‘old idea’ and that Indigenous ways of doing business are an original form of social enterprise [19,20]. Traditionally Indigenous people worked collectively and traded goods for a social purpose/community benefit [21]. In contrast, it is only recently that the limitations of the Western capitalist business model have been more widely recognised and alternatives, like the social enterprise model pursued [22].

Interest in social enterprises has been growing in Australia and internationally since the late 1990s [13,23,24]. There are different explanations for this increase; one is that social enterprises tackle social and environmental issues in a more innovative and sustainable way than other business models [15,25]. Another is the global financial crisis and its impact on governments to do ‘more with less’ [23]. Rather than governments providing services and support, they outsource to not-for-profits and social enterprises to deliver services [13,26,27]. This perception challenges the notion of social enterprises as a ‘good’ way of doing business, viewing them instead as a means for neo-liberal governments to abrogate responsibility for providing essential services [28,29].

In some ways, the emergence of the social enterprise model can be seen as a repackaging of the community business model known as the ‘community enterprise’ or cooperative, that was common in the 1970s [13,30]. Since the late 1980s not-for-profits have been exploring how the community enterprise mechanism could enable them to fulfil their mission and reduce financial dependence on government [13]. Over time there has been an increasing emphasis on trading to fulfil a mission and measuring impact and effectiveness to secure government and philanthropic funding [10,31]. The literature suggests that social enterprises exist on a continuum, ranging from enterprises with high levels of independence, to not-for-profits or community organisations that incorporate some business aspects but remain reliant on government funding [14,32]. Within this continuum Indigenous social enterprises are often more closely aligned to earlier cooperative models and to have community ownership, decision-making and governance than other social enterprise models [13,14].

The literature also delineates between social enterprises and social entrepreneurship [33,34]. Although social enterprises and social entrepreneurship both blur the boundary between not-for-profit activities and commercial activities, there are important differences between the two [33]. Social entrepreneurship is associated with social enterprise, but the term social enterprise refers to the characteristics of an organisation, while entrepreneurship is a process or action related to identifying opportunities, being innovative and taking risks [33,35]. Therefore, while social entrepreneurs seize market-based opportunities for a social purpose their business may not involve a physical entity or premises.

Overall, the literature highlights the presence of a range of different Indigenous social enterprise models according to varying degrees of Indigenous ownership, management and control [10,11,12,13,14,32]. Undertaking a scoping review to ‘scope out’ the extent of information about these models and their underlying contextual and cultural drivers may provide insight on which models are more likely to have a positive impact on Indigenous health and wellbeing than others.

## 2. Objective and Methods

The overarching objective of the scoping review was to characterize the spectrum of Indigenous social enterprises in Australia, New Zealand, Canada and the United States and identify the different operational models and cultural values that underpin the mission and activities of the social enterprises and the degree of evidence of their impact on Indigenous health and wellbeing.

This research is underpinned by ‘Indigenous Standpoint Theory’ developed by Foley [36] and Nakata [37], which challenges the power imbalance and inherent biases of Western knowledge systems [36,37,38]. Foley’s work builds on Rigney’s [38] which emphasized the importance of privileging Indigenous voices in Indigenist research and West’s [39] Japanangka paradigm that illustrates the complexities involved in navigating different worldviews [36]. According to Foley, Indigenous Standpoint Theory is more than just a research paradigm or perspective it is “a process that enables Indigenous researchers to act within their Indigenous ‘space and place’.” [36] (p. 49).

Indigenous research methodologies emphasize the importance of positioning yourself in relation to the research in order to provide transparency and context [40]. In this regard, one of the authors is a Koori (Gai-mariagal/Wiradjuri) man, while the two other authors are non-Indigenous.

Undertaking the process of Indigenous Standpoint Theory involved upholding its principles by privileging Indigenous voices in the conduct of the review and recognising the enduring legacies of colonialism. This meant taking direction from Indigenous people, most notably the Indigenous co-author and Indigenous advisory group [41]. The Indigenous advisory group is comprised of a mixture of academics and social enterprise practitioners, three are Indigenous Australians, one is Māori, and one is African American/Native American. The advisory group has met three times and provided guidance and direction in the understanding, analysis and framing of the findings of the scoping review. The scoping review methodology is not commonly associated with Indigenous research methodologies, as a result, to adopt an Indigenous Standpoint approach to the scoping review, more weight was placed on articles that had Indigenous authors and/or illustrated an awareness and knowledge of Indigenous cultural values. Adopting an Indigenous Standpoint approach, also meant that the analysis considered the structural factors and power imbalances that continue to contribute to Indigenous disadvantage.

The scoping review followed Arksey and O’Malley’s six-stage methodological framework [42] with recommended enhancements by Levac et al. [43]. Each stage is outlined below. As the scoping review progressed, some deviations to the research questions were made. The original questions did not include the values underpinning the operation of Indigenous social enterprises. However, an initial scan of the abstracts and full-text articles identified that the cultural values of Indigenous social enterprises are an important feature that distinguishes them from non-Indigenous social enterprises. The benefit of undertaking a scoping review rather than a systematic review, is that systemic reviews tend to have a narrower and more specific focus, while the more iterative nature of a scoping review lends itself to adjustments of this kind [43]. The review protocol was not registered.

### 2.1. Stage 1: Identify Research Question

In seeking to characterize the spectrum of Indigenous social enterprises in Australia, New Zealand, Canada and the United States the scoping review addressed the following research questions:

1. What are the mission and activities of Indigenous social enterprises?
2. What are the different operational models of Indigenous social enterprises?
3. What are the key characteristics of Indigenous social enterprises?
4. What are the cultural values of Indigenous social enterprises?
5. What is the relationship between the operational models, the key characteristics, and cultural values of the social enterprises?
6. What evidence is available on the impact of Indigenous social enterprises on Indigenous health and wellbeing?

### 2.2. Stage 2: Identify Relevant Studies (Literature)

A comprehensive search of the peer review and grey literatures was undertaken with support from a faculty research librarian. Four databases known to contain relevant Indigenous enterprise material were used: Scopus; Web of Science; Proquest; and Academic Search Complete/Ultimate. Other sources used were, Google Scholar, Google, and four websites (Indigenous HealthInfoNet; Office of the Registrar of Indigenous Corporations (ORIC); APO—Analysis and Policy Observatory and the Harvard Project) that are repositories for information on Indigenous organisations and enterprises. The reference list of articles included in the review were also scanned in case any relevant articles were missed during the database/internet searching process.

The search strategy was guided by two main search strings that: identified (1) Indigenous peoples in the countries of Australia, Canada, New Zealand and the USA; and (2) social enterprises. An example of the search terms used for one database is below. This search strategy was adapted when searching other databases and websites depending upon their search functions and filters:

(Aborig* OR Indige* OR Inuit OR Mäori OR “Native American” OR “American Indian” OR “North American Indians” OR “First Nations” OR “First Peoples” OR “Torres Strait”) AND (“Social Enterp*” OR “Social Business” OR “Social Entrepreneur*” OR “Social firm” OR “Community Enterprise” OR “Community Business” OR “Affirmative Business” OR “Economic Development Corporations” OR “Community Economic Develop*” OR “Community innovation”).

The search terms used for searching Google Scholar and Google were also adapted to ensure that the results did not contain too many irrelevant sources. Following Pham et al. recommendations, only the first 10 pages of search hits, comprising 100 records (10 records per page) were searched [44].

### 2.3. Stage 3: Study Selection

The third stage of the scoping review involved selecting studies based on specific inclusion and exclusion criteria relevant to the research questions. The countries of origin for the publications were restricted to countries with similar settler/colonial histories as Australia, such as New Zealand, Canada and the United States. The date range selected was 1998 to July 2021 (when the search was conducted), as it was in the late 1990s that interest in social enterprises in Australia and internationally grew [13,23]. As per Levac et al.’s recommendation the study selection was an iterative process, carried out by a team of reviewers (the three authors) who reviewed the abstracts and full text articles and met to discuss issues and uncertainties [43].

Table 1 outlines the inclusion and exclusion criteria used in the scoping review. All six inclusion criteria had to be met for a document to be included in the review.

### 2.4. Stage 4: Charting the Data

Studies that met the inclusion criteria were reviewed using Arksey and O’Malley’s descriptive-analytical narrative method [42]. A data-charting (extraction) tool was developed to record descriptive information from each document. Variables of interest included country, publication year, document type, methodology, discipline, and whether the document referred to a theory, model or framework. The charting tool also included information on the organisational characteristics of Indigenous social enterprises. Here, the classifications of social enterprises developed by Barraket et al. [13] and the Australian and New Zealand Standard Industrial Classification (ANZSIC) informed the key variables used to categorise the different social enterprises, specifically, scope, geographic setting, management structure, funding source and types of activity. The extraction tool was piloted by two reviewers with a selection of ten articles, and revisions were made to some of the variable options and categories as a result. The tool featured open-ended fields to extract descriptive and qualitative information from the documents to identify key dimensions that supported the operational models, key characteristics and cultural values of the social enterprises (see Supplementary File S1 for extraction tool).

### 2.5. Stage 5: Collating, Summarizing and Reporting the Results

The results were collated and analysed according to the three-step process suggested by Levac et al. [43].

#### 2.5.1. Stage 5 (a) Analysing the Data

Descriptive information of the documents and key variables in relation to the social enterprises (mission, activities and ownership/management structure etc…) were summarised in Excel using frequencies and percentages (questions 1 and 2). Qualitative data in relation to key characteristics or themes of the social enterprise (question 3) were inductively identified in the text and coded using the word frequency tool in NVivo. Reference to Indigenous cultural values (question 4) was also inductively identified by searching the documents for references to cultural values and assessing the context and meaning behind their use. The cultural values were recorded in the extraction tool and then analysed for common themes with the help of the Indigenous advisory group. Analysis of the relationship between the operational models and the key themes and cultural values of the social enterprises was also undertaken with the help of the Indigenous advisory group (question 5). To answer question 6, the documents were assessed to identify their methodology and whether they referred to evaluation (outcome, process), or measuring the impact or performance of Indigenous social enterprises, particularly in relation to health and wellbeing outcomes.

#### 2.5.2. Stage 5 (b) Reporting the Results

The findings were grouped in relation to each of the six research questions. A conceptual framework of the different operational models and the extent to which they articulated the key characteristics and cultural values of Indigenous social enterprises was also developed

#### 2.5.3. Stage 5 (c) Applying Meaning

The findings were considered in relation to the overarching research objective (Indigenous health and wellbeing); the theoretical underpinning (Indigenous Standpoint Theory) and the broader context of Indigenous self-determination, health equity and innovative forms of service delivery.

### 2.6. Stage 6: Consultation Exercise

The initial findings of the scoping review were shared with the Indigenous advisory group, who provided insights on the interpretation of the data and recommendations in relation to the conceptual framework.

## 3. Results

**Figure 1:**
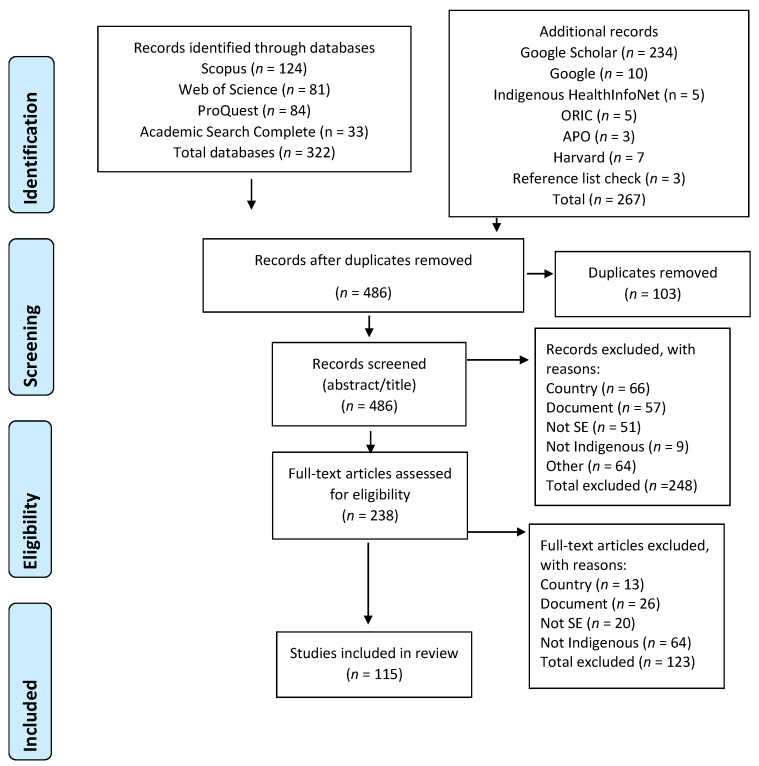
PRISMA Flowchart results.

A PRISMA flowchart depicting steps of the document identification, screening, eligibility and selection process is illustrated in Figure 1. A total of 589 documents were identified from the four databases, internet sources and reference lists of included documents, 103 duplicate documents were removed, and the remaining 486 documents were screened according to the eligibility criteria listed above. Following a review of the title and abstracts 238 documents were included in the full-text review. After screening of the full text documents 115 met the inclusion criteria (see Table S1 in supplementary materials for an alphabetical list of the documents and their sources).

### 3.1. Descriptive Characteristics of Included Documents

As outlined in Table 2, the majority of documents were about Indigenous social enterprises in Canada (42%), followed by documents about Indigenous social enterprises in Australia (28%) and New Zealand (25%). Only four publications were about Indigenous social enterprises in the United States. Most documents were published after 2014 (*n* = 78 or 68%), which shows the increasing interest in Indigenous social enterprise in recent years [45].

Just over half (54%) of the documents were peer-reviewed articles and 33% were non-peer reviewed. The majority of publications utilized empirical methodologies (68%), with qualitative methods being the most common (50%), followed by mixed methods (17%). No articles used only quantitative methods. All the reviews, except for one, were non-systematic reviews. The one systematic review of social enterprises was on all forms of ‘hybrid enterprises’ rather than just Indigenous social enterprises [18]. This systematic review also did not follow the PRISMA guidelines.

The discipline of the authors of the documents varied, but the most common disciplines were business and management (35%) followed by social science (17%), then environment and planning (15%) and community/economic development studies (14%). Only 1% of the authors came from a health discipline.

Over half (57%) of the documents mentioned a theory or theories, 82% referred to a model or models and 70% mentioned a framework. The most common types of models referred to in the literature were social enterprise models, followed by community development models, governance models and hybrid business models. The terms model and framework were sometimes used interchangeably in the literature, for example, the social economy framework and social economy model. However, they are different with frameworks tending to be more descriptive and less abstract than models [46]. This was the case in the reviewed literature with the discussion on social enterprise models broader and more high-level than the discussion on frameworks, which was more detailed and diverse. Many frameworks discussed were Indigenous specific, such as the Kaupapa Māori framework and the CREE framework [15,20,47]. The articles referring to Indigenous frameworks were also more likely to have been written by Indigenous authors and to refer to Indigenous cultural values and perspectives.

Not included in the table is the finding that half of the documents referred to social enterprises from a mixture of geographical regions (50%), followed by remote (18%), rural (15%) and urban (10%). The mixed geographical findings were reflected in the scope of the articles, with 34% of the documents about Indigenous social enterprises from a whole country or more than one country (6%). However, the majority of documents were about one or a few social enterprises from a community (40%) or region (29%).

### 3.2. Question 1: What Are the Missions and Activities of Indigenous Social Enterprises?

The Indigenous social enterprises were classified in terms of their mission and activities as outlined in Table A1 in Appendix A. The most common mission of Indigenous social enterprises was supporting community development (31%). Closely followed by providing employment and alleviating poverty (29%). Only a few social enterprises had an explicit health, wellbeing or rehabilitation purpose (4%). However, a large proportion of the documents referred to multiple social enterprises, so it was not possible to determine just one mission for them (35%).

Types of activities undertaken by the Indigenous social enterprises were diverse, ranging from agriculture, forestry and fishing (6%) to mining (1%) and wholesale and retail trade (1%). Hospitality and tourism were the two most common activities of Indigenous social enterprise, both comprising 7% of the activities of the social enterprises that were able to be identified. Adding to the complexity in mapping the types of activities was the fact that many of the social enterprises were engaged in a range of activities and so it was difficult to determine their main activity (45%). The range of activities of the Indigenous social enterprises highlights their diversity and the difficulty in trying to pigeonhole activities into a narrow range of industry classifications based on Western business models (such as the Australian and New Zealand Standard Industrial Classification). Other activities not captured by industry classifications, included:

- Passive income from leasing land
- Virtual reality to build cultural connectedness
- Traditional medicine
- Cultural healing for communities
- Cultural awareness for non-Indigenous communities
- Bushfoods

Social enterprises whose mission involved community development were more likely to be involved in multiple activities, so it was difficult to categorize just one activity (*n* = 18) (see Table A2 in Appendix A, which highlights the relationship between the mission of the social enterprises and their core activities). The second highest activity for these enterprises was tourism (*n* = 6). The types of activities undertaken by social enterprises whose primary mission was employment and poverty alleviation were more diverse and included arts and recreation services (*n* = 5), agriculture, forestry and fishing (*n* = 4), computer services (*n* = 1), mining (*n* = 1), trade (*n* = 1), tourism (*n* = 2), hospitality (*n* = 3) as well as multiple activities that could not be neatly categorized (*n* = 13). Not surprisingly, those social enterprises with an explicit link to health and wellbeing, had activities related to health care and social assistance. However, the concept of health is broad and there was one social enterprise whose mission was wellbeing that provided computer services [48].

### 3.3. Question 2: What Are the Operational Models of Indigenous Social Enterprises?

Table 3 lists a number of variables, such as the impetus for the establishment of the social enterprise and the ownership, management and funding source of the social enterprise, that were used to help determine the degree of Indigenous autonomy and control of the different social enterprises. However, many of the documents did not provide that information. Of those publications that provided detail on the ownership and management of the social enterprise (s) just under 50% were initiated by Indigenous people, 50% were Indigenous owned and 43% were Indigenous managed or governed. The majority of publications mentioned funding (77%) and in general most of the social enterprises received funding from a combination of different sources (52%).

**Figure 2:**
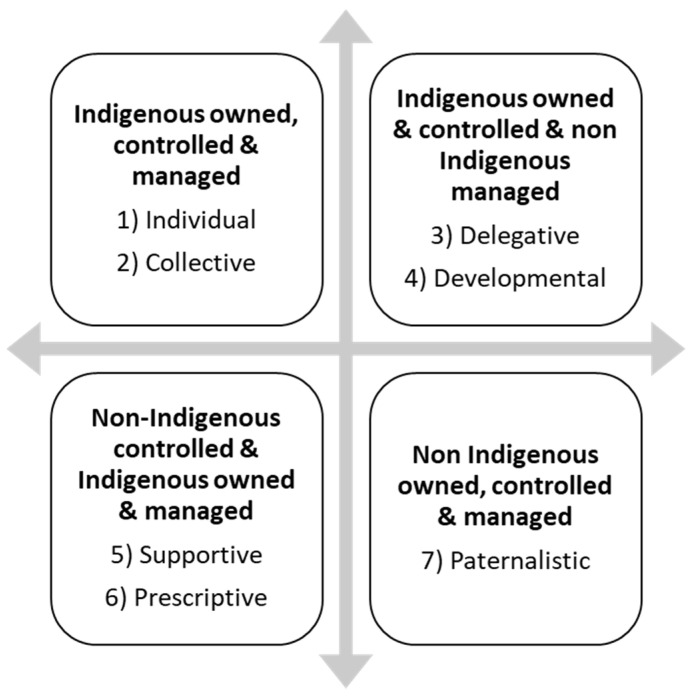
Models of Indigenous Social Enterprise based on degrees of Indigenous ownership, control and management.

Analysis of documents that contained descriptions of the operation of Indigenous social enterprise (*n* = 63 or 55%) identified four broad models based on the extent to which the Indigenous social enterprises were Indigenous owned, controlled and managed. These four broad areas were then further categorised to form seven different models: see Figure 2.

Social enterprises in the top left quadrant have the greatest degree of autonomy, followed by those in the top right quadrant. There is a question whether those in the bottom right-quadrant (non-Indigenous owned and non-Indigenous managed), should be considered an Indigenous social enterprise or not. However, examples of these models exist in the literature. There is a difference between managed and controlled. Managed refers to the day-to-day operations and the role of a CEO or manager, whereas controlled, refers to the decision-making authority of the owner, which could be an individual, board or council. Control also refers to the degree to which the social enterprise is dependent on outside funding sources, such as government or philanthropic funding.

The different organizational and legal structures of the social enterprises also influence the degree of control that Indigenous people may have. For example, whether the social enterprise is individually owned (sole trader/company) or collectively owned (an association, cooperative, partnership, company, other incorporated entity, and public benevolent institution). In a company or partnership model, if there are more non-Indigenous directors or partners than Indigenous, then there may be an imbalance of power and the Indigenous directors or partners may not have decision making authority. The organisational structure of the social enterprise can also influence where on the continuum the social enterprise sits, ranging from more socially minded social enterprises, such as traditional charities or not-for-profit organisations to conventional businesses or firms that have a social purpose but are more economically focused [49]. Examples of individually owned and managed Indigenous social enterprises (Model 1) in the literature include Indigiearth, an Indigenous Australian social enterprise, which helps Indigenous communities set up wild harvesting businesses and purchases produce back from those communities [50].

Collectively owned and managed Indigenous social enterprise (Model 2) include enterprises that Colbourne terms “high embedded” [51] (p. 118). High embedded Indigenous social enterprises have 100% Indigenous ownership, and a high percentage of their customers are Indigenous. The main mission of these social enterprises is addressing their community’s socio-economic needs and a common motto is: “for and with community.” [51] (p. 119). The extent to which a collectively owned Indigenous social enterprise is “embedded” in community varies depending on the socio-political context of the enterprise [51].

Indigenous owned and controlled and non-Indigenous managed social enterprises can be divided into two types, “delegative” (Model 3) or “developmental” (Model 4). In the delegative model the Indigenous owners (board or council) choose to appoint a non-Indigenous manager for political or strategic reasons [11]. In the developmental model the non-Indigenous manager is usually appointed because there is no Indigenous person in the community with the requisite skill set (literacy and numeracy) to be a manager. The developmental model is more of necessity than a choice and is particularly common in remote areas, where local Indigenous people have often been denied the educational opportunities to manage their own enterprises. For example, the Nuwul Environmental Services in Arnhem Land in Australia is owned by the Rirratijingu clan but managed by a non-Indigenous manager [17,52,53]. Indigenous social enterprises that are developmental models can transition to become collective models’ overtime. For instance, Wandjina Tours in Australia was initially established as a partnership between an Indigenous director and a non-Indigenous director and overtime it transitioned into a wholly Indigenous owned and operated social enterprise embedded in community [54].

Indigenous owned and managed and non-Indigenous controlled enterprises include models where most of the funding comes from outside sources, such as government or philanthropic funding and can be further broken down to the “supportive” model (Model 5) and the “prescriptive” model (Model 6). The supportive model is when the funder supports the Indigenous manager or Indigenous council to deliver activities and run the social enterprise in line with Indigenous cultural values. For example, Tjanpi Desert Weavers’—a social enterprise of the Ngaanyatjarra Pitjantjatjara Yankunytjatjara (NPY) Women’s Council, helping Indigenous women earn an income from contemporary fibre art. Tjanpi Desert is funded by Caritas Australia, an international aid and development organisation of the Catholic Church but governed by the NPY Women’s Council [55].

The prescriptive model is when the funder (often government) determines what approach the social enterprise will take in the delivery of activities and the running of the social enterprise [28]. Often these types of enterprises were traditionally not-for-profits but have become social enterprises to compensate for a decrease in government funding. Their reliance on government funding has also contributed to the view that Indigenous social enterprises are unsustainable or unbusinesslike [21]. This model is also used as an example of how neo-liberal policies have negatively impacted the not-for-profit sector [28,29]. The difference between this model and the “supportive” model is the degree of respect shown to Indigenous cultural values and governance. The “prescriptive model” is more likely to control how funding is used and to impose Western business practices on the operation of Indigenous social enterprises [56]. As a result, a number of Indigenous social enterprise practitioners avoid applying for government funding so that they have the freedom to operate in way that best suits their community [28].

The non-Indigenous owned, controlled and managed, ‘paternalistic’ model (Model 7) is sometimes considered Indigenous (even though it technically is not) because its mission is to support Indigenous people in some way. This model includes examples of social enterprises that have been started by non-Indigenous not-for-profits, who have ‘partnered’ with an Indigenous Community Controlled Organisation and which are managed by a non-Indigenous manager [57,58]. If the partnership arrangement is only tokenistic, then the degree of Indigenous control and ownership is negligible. In Canada social enterprises led by non-Indigenous people serving Indigenous and non-Indigenous people are the most prevalent social enterprise model supporting Indigenous people [59].

### 3.4. Question 3: What Are the Key Characteristics of Indigenous Social Enterprises?

Inductive and content analysis of the documents using NVivo identified five key characteristics or themes of Indigenous social enterprise, as outlined below:

- Self-determination and how this related to ownership, control, governance and self help
- Sustainability—across multiple spheres, economic, social, cultural, community development and impact
- Innovation—inventive, new, or creative/original approaches
- Value and or values, and how this relates to different types of values, such as social value and cultural values, as well as value chains and frameworks that measure value.
- Hybridity—this referred to blending across social and economic objectives and combining Western and Indigenous perspectives.

These characteristics or themes are discussed below in relation to the models presented above in Figure 2.

#### 3.4.1. Self-Determination

The extent to which the models supported Indigenous self-determination varied and tended to decline as the level of Indigenous ownership, control and management of the enterprise diminished. For collectively owned (embedded) social enterprises, self-determination was often the principal objective behind the establishment of the social enterprise [12,60,61,62]. In many communities the social enterprise is a symbol of Indigenous self-determination and a way for communities to achieve emancipation from the state [63]. For example, Anderson et al. [12], states that Indigenous communities in Canada are pursuing social enterprises to obtain greater control of activities on their traditional lands, increase their economic-self-sufficiency and preserve and strengthen their traditional cultural values. An example of a Canadian Indigenous social enterprise is the Osoyoos Indian Band Development Corporation (OIBDC). The corporation’s motto is: “working with business to preserve our past by strengthening our future” [12] (p. 52). The mission of the corporation is for the community to become self-sufficient and decrease their dependency on government funding. Through a range of business ventures, including a construction company and a winery, the community is hoping to not only improve members educational and employment outcomes but to also strengthen culture, by reinstalling traditional concepts of honour, caring, sharing, and respect [12].

At the same time, while there are many positive examples of collective social enterprises delivering self-determination, there were also examples where the governance arrangements of Indigenous social enterprises prevented this from occurring [64]. In one community, the chief and Council were successful in establishing a community enterprise, but they were not successful in cultivating entrepreneurship and self-sufficiency among the broader community [64]. The reason given for this was because the chief and the Council did not involve the community in their decision making or provide them with any opportunities to learn or practice entrepreneurship [64]. The involvement of external partnerships may have also been a factor [64].

#### 3.4.2. Sustainability

All the different models referred to sustainability in some way, though it was articulated differently across each of the seven models. While sustainability can mean the long-term viability of the enterprise for collectively owned enterprises, sustainability was associated with the environment, such as ‘Caring for Country’ [15,65,66] and the survival of culture as well as sustainable business practices. Indigenous notions of sustainability are different from Western concepts, which are often focused on what resources people can continue to take without causing ‘too much’ harm to the environment [67,68]. Sustainability from an Indigenous perspective embodies gratitude for what nature gives and emphasizes caring for the environment rather than simply reducing the amount taken [67]. Several of the collectively owned and embedded Indigenous social enterprises reflect this cultural obligation of custodianship and stewardship in the way they operate [15,69].

An example of Indigenous sustainability practices is a social enterprise in New Zealand, which places limits on the growth and development of the enterprise to protect the local penguin population [15]. The social enterprise, Blue Penguins Pukekura (BPP) is a small tourism operation providing visitors with nightly wildlife tours of the Little Blue Penguin. The social enterprise successfully balances the Māori’s community’s cultural obligation to care for the environment alongside their responsibilities to provide a sustainable business model for future generations. In order to ensure the survival of the Little Blue Penguins, the enterprise closes public access to the beach at certain times (such as breeding season). As one of the employees of the social enterprise noted “the business model could collapse [if] the penguin population collapses” [15] (p. 489). To minimise the negative impact on the business, widespread communication with key stakeholders was conducted [15].

#### 3.4.3. Innovation

The individually owned Indigenous social enterprise model is strongly associated with innovative and creative ways of doing business. An example is iMOKO^TM^ a software app used in Kōhanga Reo, early childhood centres and schools in Far North New Zealand, where access to health services is limited [20]. At each centre, a volunteer or teacher’s aid is trained to help parents submit information about their children’s ill health to a team of telehealth clinicians. The clinicians assess the information and make a recommendation to a doctor for review. The doctor reviews the case, makes any necessary adjustments to the diagnosis, and then creates a script. The script is then sent to a pharmacy near where the patient lives for collection [20]. The iMOKO^TM^ social enterprise’s innovation is in how it disrupts power relations. By putting access to healthcare into the hands of parents, caregivers, and the broader community, it shifted the power away from the government provided health services and is contributing to “systemic social change” [63] (p. 787).

Collectively owned social enterprise can also be innovative in the way they operate to bring about cultural revitalization and social change [70,71]. For example, a community owned enterprise in East Arnhem Land in Australia is using government funding from an unemployment scheme and probation work orders to increase its number of employees [17]. The funding from these government employment programs enables the enterprise to employ more people than needed, which means there is the flexibility for staff needing to take leave to meet their cultural obligations, but still enough employees for the enterprise to meet all its business needs. In this instance the social enterprise is subverting the government’s restrictive and culturally disempowering unemployment program (which has strict attendance requirements) and is creating a more empowering and culturally appropriate employment model [17].

#### 3.4.4. Social Value

Like many social enterprises, most of the different Indigenous social enterprise models contribute to social value in some way by creating jobs and strengthening social capital [72]. However, the strengthening of social capital was more apparent in the collective, delegative, developmental and supportive models, where Indigenous cultural values are supported than in the prescriptive or paternalistic models. Social capital is defined as: “a collective asset in the form of shared norms, values, beliefs, trust, networks, social relations, and institutions that facilitate cooperation and collective action for mutual benefits.” [73] (p. 480). Within the Indigenous social enterprise sector, contributing to social value is strongly linked to strengthening cultural values and improving the overall health and wellbeing of people and communities.

For example, a collectively owned social enterprise in Australia illustrates how the cultural values of sharing and reciprocity can play a significant role in a social enterprise’s operation [52]. The enterprise has reciprocal arrangements with other local organisations in the community to borrow vehicles and large landscaping equipment when completing multiple contracts simultaneously. In return, the enterprise stores its big equipment and vehicles at the local council’s property, so they are available for other local organisations to use if needed. While there are some risks that the vehicles may not always be available when needed, this arrangement allows the social enterprise to increase its profit base and grow the business, rather than spending its profits purchasing depreciating assets [52]. It also has a positive impact on relationships within the community between Indigenous and non-Indigenous people [52].

The social value of Indigenous social enterprises is also reflected in the opportunities they provide community members to connect with their culture and heal. For example, Bana Yarralji Ltd., a collectively owned Australian Indigenous social enterprise provides cultural camping on country experiences [66]. The goal of this social enterprise is to “heal family and community both spiritually and physically through connection with the land.” [74] (p. 38).

#### 3.4.5. Hybridity

All social enterprises are hybrids to a certain extent because they combine social needs with business responsibilities. However, Indigenous social enterprises not only do this, but also combine traditional cultural values and practices with Western business methods [17,25]. While hybrid enterprises were common within the collective model, hybridity was also a feature of many of the other models (notably, individual, delegative, developmental and supportive). A social enterprise in Canada is KO-KNET, a self-controlled and self-owned community model for digital infrastructure, which has taken control of the local internet infrastructure [48]. KO-KNET resists the notion that ‘traditional’ (Indigenous) and ‘modern’ are opposing categories [48]. The blending of Western and Indigenous knowledge is reflected in the mission of several social enterprises, which emphasize the importance of cross-cultural learning and doing things ‘two ways’ [52]. The notion of a ‘hybrid economy’ is particularly visible in remote parts of Australia, where many Indigenous people are engaged in activities like wildlife harvesting as a form of social enterprise [21,75]. Although, the extent to which these wildlife enterprises apply conventional business practices varies, they are a form of social enterprise in the traditional sense, as their principal concern is providing a social good by meeting the needs of the community [75]. 

### 3.5. Question 4: What Are the Cultural Values of Indigenous Social Enterprises?

The review identified that 82% of the documents referred to cultural values, highlighting the important relationship between cultural values and Indigenous social enterprises. A number of documents also stated that it was this cultural element that was the key distinguishing feature between Indigenous social enterprises and social enterprises in general [14,51,60]. Cultural values were identified inductively across the literature. With the help of the Indigenous advisory group similar words were grouped together into the following seven categories:

1. Foster/support/strengthen/uplift (particularly in relation to culture and empowerment)
2. Protect/stewardship/guardianship/treasure/care for/heal (particularly from trauma)
3. Share/reciprocity/hospitality/generosity
4. Relationality—the importance of relationships/family/kinship
5. Survival/resilience importance of humour as a survival mechanism
6. Respect/honour/etiquette/protocol or “way”
7. Unity—(bring tother, unite and wholeness/holism).

Cultural values were reflected in the mission and activities of Indigenous social enterprises as well as in the way the social enterprises operated. For example, the mission of a number of social enterprises was to support the maintenance and protection of traditional practices—such as weaving, land management, collecting bushfoods and hunting [21,55,65]. Yet, even when social enterprise’s activities were not directly related to traditional practices, cultural values still influenced the way the social enterprises operated, from the governance arrangements of the organisation to the way in which the social enterprises worked with and supported the community. For example, humour was used as a way of managing relationships and ensuring the survival of the social enterprise [69,76]. What traditional businesses might consider expenses and something to be minimized (such as salaries, staff training and development), were seen by many Indigenous social enterprises as opportunities to deliver socioeconomic benefits to their communities and improve health and wellbeing [65].

Although Indigenous cultures are diverse, there are commonalities. In general, Indigenous people tend to prioritise “holistic well-being and value creation over profit maximization” [77] (p. 465). In the Māori entrepreneurial world view, there is the notion of the Takarangi spiral of innovation, a double spiral, linking spiritual and human ancestors with descendants not yet born—balancing tradition with opportunity [78]. A similar concept is found among the Canadian Indigenous people, who view sustainability as ensuring the survival of the people, the land and the resources for seven generations [79]. In Australia, many Indigenous people have a deep connection to Country and “…success is not measured in terms of tangible assets, but in the pluralism of familial relationships, religion, and spiritual connections with the landscape.” [75] (p. 52).

### 3.6. Question 5: What Is the Relationship between the Operational Models and the Key Themes and Cultural Values of the Social Enterprises?

Table 4 is an integrative conceptual framework of Indigenous social enterprise, which maps how the seven different social enterprise models reflect the key themes and cultural values identified in the literature, particularly those documents that contained descriptions of the ownership, management, and funding arrangements of the Indigenous social enterprise (*n* = 63 or 55%). The most common model across the literature was the collective model (*n* = 53), followed by the individual model (*n* = 15), the developmental model (*n* = 8) and the delegative model (5). There was less support in the literature for the supportive, prescriptive, and paternal models (*n* = 3 for each). Yet, while the numbers were small, each of these models had clear distinguishing features that set them apart from the others.

Each of the models articulate the key themes and cultural values in different ways depending on the level of Indigenous ownership, control and management of the social enterprise. Models that are empowering and allow for more self-determination, are more likely to incorporate a range of cultural values (like reciprocity and sustainability) and to foster cultural strength and provide healing, than models that are prescriptive or paternalistic. Several of the cultural values apply to more than one of the five different key themes or characteristics, illustrating how cultural values can be applied in different ways depending upon the circumstances. For example, protect is both a social value and a means of achieving sustainability. Reciprocity is important for sustainability, but it is also a key aspect of hybridity which relies on sharing different perspectives for mutually beneficial outcomes.

### 3.7. Question 6: What Evidence Is Available on the Impact of Indigenous Social Enterprises on Indigenous Health and Wellbeing?

While evaluation was mentioned in 37% (*n* = 43) of the publications, there was very little empirical evidence on the impact of social enterprise on Indigenous health and wellbeing. Only one article referred to the evaluation of Indigenous social enterprises in relation to health and wellbeing services, and this article was not specific to just Indigenous social enterprise [80]. Furthermore, although 37% of the documents referred to evaluation in some way, very few of them were an actual evaluation. Only 13 (11%) of the peer-review articles referred to evaluations of Indigenous social enterprises and none were actual evaluations. Most of the evaluations were in the grey literature and tended to use non-Indigenous evaluation methods, with some noticeable exceptions from New Zealand [81,82,83,84]. These evaluations provided some evidence of the impact of Indigenous social enterprises on health and wellbeing. For example, two enterprises in New Zealand were food trucks providing healthy food to Māori communities and there was some evidence that this was having an impact on peoples’ health [82].

However, although there were few evaluations, there were some examples of Indigenous social enterprises supporting peoples’ health and wellbeing. For example, two social enterprises in New Zealand “Patu Aotearoa” and “Toa Fit” aimed to get Māori active and healthy [20]. There were also many implicit connections linking Indigenous social enterprises to beneficial health outcomes as most Indigenous social enterprises aimed to improve the overall wellbeing of their communities by alleviating poverty through employment and community development activities and by strengthening cultural connections.

## 4. Discussion

The scoping review identified a number of non-systematic literature reviews of Indigenous social enterprises (21%); however, this is the first scoping review to comprehensively and systematically examine Indigenous social enterprises in Australia, New Zealand, Canada and the United States. While several other systematic reviews have highlighted the importance of Indigenous self-determination and cultural values in health governance models and health and wellbeing [85,86] no other systematic review has looked at how Indigenous social enterprises impact on Indigenous health and wellbeing.

The scoping review identified the different characteristics and operational models of Indigenous social enterprises and the relationship between the mission and activities of these enterprises. The range of activities undertaken by the different Indigenous social enterprises, highlights their diversity. Indigenous social enterprises are not just in the agricultural, tourism, arts and training sectors but also involved in mining computing and viticulture industries.

A comprehensive conceptual framework featuring seven models of Indigenous social enterprises based on differing levels of Indigenous ownership, control and management: (1) individual, (2) collective, (3) delegative, (4) developmental, (5) supportive, (6) prescriptive and (7) paternalistic; in relation to five themes (self-determination, sustainability, innovation, social value and hybridity) and seven cultural values (1) Foster, (2) Protect (3) Reciprocity, (4) Relationality, (5) Survival (6) Respect and (7) Unity was developed (Table 4). However, the seven cultural values identified, are not easily defined in one word. Often there is no direct translation from the Indigenous word into English, for example the Māori word tikanga is described in English as values, rules, priorities, and ways of doing things [22]. Furthermore, while there are broad similarities across the different countries, each Indigenous cultural group has their own interpretation of the cultural values. This finding is similar to a recent systematic review on Indigenous conceptualizations of wellbeing, which found that although there are commonalities, Indigenous peoples’ values and conceptions of wellbeing are shaped by their experiences and local contexts [86].

Models with a strong degree of Indigenous ownership, control and management were more likely to embody Indigenous cultural values through their mission, activities, and operations than those that were managed, controlled or owned by non-Indigenous people. The Indigenous cultural values that underpin the operation of Indigenous-led social enterprises are helping to strengthen cultural connections and heal their communities, both spiritually and physically [74]. Though, the prescriptive and paternalistic models did refer to cultural values, the way these values were articulated in the day-to-day operations of the enterprises tended to be quite transactional or tokenistic [56,57,58].

Despite the lack of evaluations providing explicit evidence of the impact, it was apparent that many Indigenous social enterprises aim to implicitly improve Indigenous peoples’ health and wellbeing through their missions and activities and the embodiment of Indigenous cultural values. Even though only a small percentage of Indigenous social enterprises are directly involved in providing health related activities and services (4%), the broader mission of the social enterprises to alleviate poverty and support community development suggests that Indigenous social enterprises could play a key role in improving the socio-economic determinants of health in their communities. The review also suggests that Indigenous social enterprises could be a more innovative way of improving health outcomes than many traditional public health programs, which are often constrained by their funding to operate in silos. By generating their own funding many Indigenous social enterprises have the flexibility to operate in a more holistic and culturally relevant way.

Social enterprises have been influenced by their historical, socio-cultural context, particularly the relationship the state has with Indigenous people. Despite the similarities they share as settler colonial states, there were notable differences between the Indigenous social enterprises from the different countries and the degree of self-determination they had.

New Zealand Māori have a different relationship with the state than Indigenous Australians due to the Treaty of Waitangi and various other historical and political reasons. Under the Treaty of Waitangi settlements Māori iwi (tribes) received significant assets from the Crown to compensate for historic injustices. These iwi-based enterprises are examples of the collectively owned and embedded social enterprise model and play an important role in New Zealand’s economy, accounting for some 5.6% of the country’s GDP [87]. The majority of Māori social enterprises are limited liability companies [20]. However, there is a distinction between the large iwi-based enterprises and smaller non-iwi Māori-led, community-based organisations and individually owned social enterprises [22]. Many of the smaller social enterprises developed by the Māori are quite innovative [63,81,88]. For example, iMOKO^TM^ which uses technology to democratise healthcare and improve the health outcomes of the Far North Māori [61], and Māori Maps, which connects urban-based Māori with marae (sacred community meeting places and ancestral homes) [88].

The majority of Indigenous social enterprises in the Australian literature were community run enterprises in rural and remote locations, dependent on government for some of their funding (the developmental, supportive or prescriptive models). However, one of the reasons for this trend, was that three of the documents were about the same enterprise in East Arnhem Land—Nuwul Environmental Services, which is in a very remote area of Australia [17,27,52]. The focus on Indigenous social enterprises in rural and remote locations reflects other literature on Australian entrepreneurship, which has been criticised for stereotyping Indigenous businesses as only being in the outback [89]. There is, however, some truth to this perception, as the lack of viable employment options in remote Australia has necessitated the need for social enterprise in remote locations [73].

Similarly, Canada’s history of supporting Community Economic Development (CED) corporations on Indigenous land probably contributed to the large number of collectively owned social enterprises discussed in the literature [12,90]. In addition to collectively owned/embedded organisations, the literature also discussed social enterprises that were corporations, multi-tiered cooperatives, and social-purpose businesses [59]. A number of Canadian social enterprises were established by non-Indigenous people but are now run by Indigenous people (the developmental model) [59]. There are also a number of social enterprises in Canada that provide services to Indigenous people that are not led or managed by Indigenous people (the paternalistic model) [59].

In the United States, the literature referred to Indigenous social enterprises as social entrepreneurship and described it as embodying a mixed strategy of traditional economy, individual market enterprise, and tribal government-managed corporations (collectively owned and embedded models) [91]. Most of the social enterprises in the United States literature were from Alaska [91,92,93]. The low number of articles about Indigenous social enterprises from the United States was surprising. However, this corresponds with another recent study which found a lack of research on Native American social enterprises [94].

The difference between the four countries, suggest that it will be important to understand how context influences the mechanisms used by Indigenous social enterprises to improve health and wellbeing in their communities. For instance, the political context can influence the degree of control Indigenous social enterprise practitioners have over decision-making. For example, in Australia, billions of dollars are spent annually on Indigenous programs to try and ‘close the gap’ between Indigenous and non-Indigenous people’s socioeconomic outcomes, with little success [95]. An argument given for the failure of the Closing the Gap policy was that it was a heavily programmatic, government-controlled response, which failed to recognize the importance of Indigenous self-determination [9]. Recently, Indigenous leaders have managed to negotiate a new Closing the Gap agreement with Australian governments that recognises the benefits of Indigenous community-controlled organisations as an act of self-determination and prioritises them to provide services to Indigenous peoples and communities [96]. Within this new political context, the time might be right to explore how Indigenous social enterprises could play a more explicit role in improving Indigenous health outcomes.

### 4.1. Implications for Future Research

Although broad conclusions about the positive impact of Indigenous social enterprises on health outcomes can be made, more explicit research directly focused on the relationship between social enterprises and health is needed. The fact that only 1% of the authors in the scoping review came from a health discipline suggests there is a gap in the research. This finding also aligns with the findings of a recent systematic review of non-Indigenous social enterprises, which found there was a lack of research on the relationship between social enterprises and health [97]. Indigenous social enterprises may offer a more holistic approach to health equity than the siloed, top-down government-controlled model currently in place [9]. However, further investigation of Indigenous social enterprises is needed to determine this, including evaluations which measure the impact of Indigenous social enterprises in more culturally appropriate ways.

### 4.2. Limitations

One limitation of the scoping review is the search strategy appears to have been biased towards collectively owned social enterprises even though the terms social entrepreneur* was used. At the same time, the search strategy did result in a large number (*n* = 115) of publications, which suggests that it would not have been practical to have widened the scope of the search strategy further. The aim of a scoping review is to ‘scope out’ the extent of information on a particular topic. In this regard, the scoping review has highlighted a broad range of literature on Indigenous social enterprise with no clear agreement across the literature on a specific definition of an Indigenous social enterprise

Another limitation is that a scoping review is a Western research methodology commonly used in the health sciences, whose systematic and rigid approach, is not necessarily aligned with Indigenous Standpoint Theory. It was difficult to challenge the inherent biases of Western knowledge systems while using the scoping review methodology. The quantitative focus of the scoping review in extracting data had to be balanced with an inductive approach that privileged Indigenous voices through the consultation process and in the analysis of the literature, which placed more weight on Indigenous authors.

### 4.3. Strengths

Conversely, one of the limitations of this study is also one of its strengths. Although it was hard reconciling Indigenous Standpoint Theory with a scoping review methodology, it did help to provide a more comprehensive understanding. A benefit of systematic literature reviews is their more rigorous approach helps to provide certainty around any gaps identified in the literature. At the same time, adopting an Indigenous Standpoint approach helped to recognise and overcome the inherent bias towards positivism in the scoping review methodology. According to Kimmerer, when combining Western methodology with an Indigenous theoretical framework, it is important to recognise the inherent differences of the two approaches and that blending them could reduce their distinctiveness [67]. Each strand must be weaved together separately to create a whole [67]. The difficulties experienced in undertaking this process also helped to provide an appreciation for the challenges that Indigenous social enterprise practitioners face in trying to integrate Indigenous values into a Western framework. In this regard the Indigenous advisory group, played a key role in providing the knowledge to apply an Indigenous Standpoint approach and navigate the two worlds.

Finally, this study is one of only a few examples of a study about social enterprise written by academics from a health faculty. Most of the social enterprise literature is written by scholars from business and management faculties or schools. As a result, the study has helped to identify an important gap in the literature, which could be investigated further by health researchers seeking to identify more equitable, culturally appropriate and innovative ways of delivering health outcomes to Indigenous peoples.

## 5. Conclusions

While there have been a number of reviews on Indigenous social enterprises, this is first scoping review to look at Indigenous social enterprises in Australia, Canada, New Zealand and the United States. It also one of only a few studies in Australia exploring the link between Indigenous social enterprises and health and wellbeing.

The scoping review found that the most common mission (Q. 1) of Indigenous social enterprises was supporting community development, followed by providing employment and alleviating poverty. Only a few Indigenous social enterprises had an explicit health, wellbeing or rehabilitation purpose and were directly involved in providing health related activities and services. However, the broader mission of Indigenous social enterprise to alleviate poverty and support community development, indicates their role in improving the socio-economic and cultural determinants of health in their communities. Indigenous social enterprises are involved in a diverse range of activities, from land-based and traditional cultural practices to computer services, mining and operating a winery

Seven different models of Indigenous social enterprises were identified (Q. 2) based on levels of Indigenous and non-Indigenous ownership, control and management of the social enterprise. Some of the different models (individually owned and collectively owned/embedded) were more likely to contribute to improved health and wellbeing outcomes by increasing self-determination and helping to strengthen cultural connections and improve health and wellbeing than others.

Thematic analysis of the literature identified five common themes (Q.3): (1) self-determination, (2) sustainability, (3) innovation, (4) social value and (5) hybridity. The relationship between these five themes and the cultural values highlights how cultural values can be applied in different ways. For example, protecting is both a social value and a means of achieving sustainability.

A key point of difference between Indigenous and non-Indigenous social enterprises is the cultural values that underpin their operation (Q. 4). The large number (82%) of articles that referred to cultural values of Indigenous social enterprise, reflects the important role that culture plays in Indigenous social enterprises. Cultural values are not only reflected in the mission or activities of Indigenous social enterprises but also in the way that the social enterprise operates. Although Indigenous cultures are diverse, there are similarities, with seven common types of cultural values identified across the literature. These included: fostering, protecting, sharing, relationality, survival, respect and unity.

Models with 100% Indigenous ownership and control were more likely to embody Indigenous cultural values in their mission and activities and contribute to improved health and wellbeing by increasing self-determination and strengthening culture and promoting healing than others. (Q.5). As a result, despite the lack of empirical evidence linking Indigenous social enterprises to improved health and wellbeing (Q. 6), Indigenous social enterprises could offer a more holistic and sustainable approach to health equity and health promotion than the siloed, programmatic model common in public health policy.

## Figures and Tables

**Table 1:** Inclusion and exclusion criteria of scoping review.

Criterion No.	Criterion Type	Inclusion	Exclusion
1	Country of study	Australia, New Zealand, Canada, USA	All other countries not Australia, New Zealand, Canada and USA
2	Date range	1998–2021	Publications prior to 1998 and after July 2021
3	Document Type	Journal articles, non-peer review reports, books and theses	Newspaper articles, opinion pieces/commentary, editorials, book reviews
4	Type of enterprise	A form of social enterprise, including community enterprise, businesses or community organisation, hybrid businesses and Economic Development Corporations (EDCs).	Individual entrepreneurs or private, for-profit businesses
5	Indigeneity of social enterprise	An Indigenous owned social enterprise, or a social enterprise designed to provide services/support to Indigenous people only	Non-Indigenous social enterprises only
6	Language	English	All other languages

**Table 2:** Descriptive characteristics of included documents (*n* = 115).

	*n*	%
**Country**		
Australia	32	28
Canada	48	42
New Zealand	29	25
USA	4	3
Multi	2	2
**Year of Publication**		
2003–2008	13	11
2009–2014	24	21
2015–2021	78	68
**Type of Document**		
Peer-reviewed article	62	54
Book chapter	9	8
Thesis	6	5
Non-peer reviewed document	38	33
**Methodology**		
***Empirical***		
Quantitative	0	0
Qualitative	57	50
Mixed method	20	17
Systematic review	1	1
***Non-Empirical***		
Commentary/opinion	5	4
Non-systematic review	23	20
Descriptive	9	8
**Discipline**		
Business/Management/IT	40	35
Community/Economic Development	16	14
Environment (sustainability/planning)	17	15
Health	1	1
Law	2	2
Social Science	20	17
Tourism	3	3
Not affiliated with a university	16	14
**Theoretical/conceptual underpinnings**		
Reference to Framework	80	70
Reference to Model	94	82
Reference to Theory	57	50

**Table 3:** Operational characteristics of Indigenous social enterprises (*n* = 115).

	*n*	%
**Impetus**		
Indigenous	56	49
Non-Indigenous	4	3
Not stated unable to determine or not applicable	55	48
**Ownership**		
Indigenous	58	50
Non-Indigenous	4	3
Not stated or unable to determine	53	46
**Management/Governance**		
Indigenous	49	43
Non-Indigenous	12	10
Not stated or unable to determine	54	47
**Funding (mentioned)**		
Yes	88	77
No	27	23
**Funding Source**		
Self-generated/enterprise	4	3
Combination	60	52
Government	2	2
Philanthropic	2	2
Resource company (mining)	1	1
Not stated or unable to determine	24	21

**Table 4:** Conceptual framework of Indigenous social enterprise (SE) and their relationship to key themes and cultural values identified in the literature (*n* = 90) ^1^.

Themes & Cultural Values	Individual Indigenous Owned, Controlled & Managed (*n* = 15)	Collective (Embedded) Indigenous Owned, Controlled & Managed (*n* = 53)	Delegative Indigenous Owned & Controlled & Non-Indigenous Managed (*n* = 5)	Developmental Indigenous Owned & Controlled & Non-Indigenous Managed (*n* = 8)	Supportive Indigenous Owned & Managed & Non-Indigenous Controlled (*n* = 3)	Prescriptive Indigenous Owned & Managed & Non-Indigenous Controlled (*n* = 3)	Paternal Non-Indigenous Owned, Controlled & Managed (*n* = 3)
**Self-determination** Foster, strengthen Survival Respect, protocol Heal	Strongly associated with self-determination & survival. The mission of the SE is often to provide employment & alleviate poverty, while strengthening cultural connections. For some the SE may be a form of healing & overcoming trauma.	Strongly connected to protocols & Indigenous ways of doing business. Often a symbol of Indigenous self-determination in their communities & the mission of the SE is often focused on strengthening community & culture to ensure its survival. At the same time, governance issues sometimes impact the achievement of this mission.	Indigenous ownership & control ensure a high degree of self-determination. Indigenous board choose to appoint non-Indigenous manager to avoid political issues & to ensure the survival of the SE. May be some differences of opinion between board & broader community.	Indigenous ownership & control ensure some level of self-determination, but cultural values may be compromised by non-Indigenous managers who do not observe/have respect for cultural protocols.	Non-Indigenous funder is respectful of Indigenous cultural protocols, can be empowering & help to foster & strengthen Indigenous culture. At the same time, community is dependent on external agency for funding, which creates uncertainty & reduces self-determination.	The degree of control exercised by the funder or non-Indigenous partner can weaken opportunities for self-determination & impact on the ability for cultural values to be incorporated into the operation of the SE, affecting cultural survival.	Limited opportunity to foster/strengthen Indigenous culture & contribute to Indigenous self-determination. Is less likely to follow cultural protocols & be respectful to Indigenous people. Cultural activities & displays may lack authenticity & feel tokenistic.
**Sustainability** Protect, treasure, stewardship, guardianship. care for, heal Survival Share, reciprocity	A key focus of these SE is sustainable business practices, which include caring for the environment, the survival of culture & the ongoing viability of their business. Sustainability is not about what you can take but what you can continue to give.	Limits on the growth & development of the SE are often applied to ensure the protection of the environment & to uphold cultural values of stewardship. Gratitude for what nature gives, rather than seeing natural resources as something to take. SE focused on restoring and healing the environment.	Many were established to realise beneficial conservation or restoration outcomes, & non-Indigenous managers are sometimes brought in for their expertise in supporting this goal.	A strong focus on traditional cultural values, however, sometimes the non-Indigenous manager can decide that while the community’s traditional way of doing things may be culturally sustainable, they are not economically sustainable, & there can be tension.	Traditional Indigenous cultural practices are recognised as more beneficial for the environment than non-Indigenous, & the funder supports Indigenous people to undertake environmental protection or Caring for Country activities, either as employees or participants of the SE.	Indigenous people are specifically funded to undertake certain activities, which may include, environmental or tourism programs. While there may be strong overlap between these activities & the traditional values of caring for Country, there is less autonomy and control.	Environmental sustainability may be a focus but there is not a high degree of cultural sustainability in this model, & if there are aspects of traditional culture, they may be lacking in authenticity.
**Innovation** Survival, humour Foster, Strengthen	Individually owned SE are strongly associated with innovative & creative ways of doing business (which may include humour). Often the SE blend traditional practices with technology as a way of ensuring the survival and strength of Indigenous culture.	Some are extremely innovative in how they blend tradition and modernity in order to survive, others may lack innovation because they are wedded to doing things in a certain way (for traditional or historical) reasons.	Assistance from a non-Indigenous manager may enable Indigenous people to participate in economic activities in novel & interesting ways. The SE may use technology to strengthen culture.	There is sometimes a degree of innovation in the way the SE is run, for example how government funding is used to ensure the sustainability of the SE and continuation of cultural practices.	The supportive model can demonstrate a high level of innovation in how it supports Indigenous communities’ aspirations for the SE. This includes the type of activities undertaken by the SE to help foster and strengthen Indigenous culture.	This model does not tend to provide the opportunity to foster or strengthen culture in an innovative way.	Some of these models can appear to be quite innovative, though not usually in a cultural way.
**Social Value** Family/Kinship Share/reciprocity/hospitality/generosity Protect/treasure/care for/heal	Although they are individually owned SE, many are strongly connected to the broader Indigenous community & the cultural values of sharing & reciprocity. A number of SE provide a % of their profits to community either directly or indirectly to help others heal/overcome intergenerational trauma.	Family & kinship responsibilities can be central to the operation of these SEs. The social value of the SE is strongly associated with the opportunities it provides to strengthen relationships through employment & other benefits.	The delegative model is often chosen as it allows Indigenous communities to separate family responsibilities from business requirements. While the SE may provide benefits through employment and or the provision of services, demand sharing practices are curtailed.	Sharing & reciprocity can play a significant role in the operation of these SEs. With the development of capacity illustrated through reciprocal partnerships.	Supportive SE can play a role in protecting & caring for culture & enhancing family & kinship relationships.	The social value can be difficult to find. The model may provide employment opportunities for family/kin, but the level of outside control can also cause conflict among family members.	These types of enterprise are often very interested in measuring social value but the social value they provide is limited. Support can come across as paternalistic & people may feel shame. Any form of reciprocity is more transactional or tokenistic ‘you get this, and I will give you this’
**Hybridity** Reciprocity Unity	Individually owned SE can blend both Western business practices with cultural values/practices. Can be viewed as a form of reciprocity as is a mutually beneficial arrangement—to both customers & the SE.	Community owned SEs are by their very nature a blend of Indigenous cultural values & Western business practices. The reciprocity lies in the sharing of cultural knowledge through the incorporation of two world views.	A blend between western & Indigenous ways of doing things, reflecting many Indigenous people’s desire to live in both worlds.	A hybrid model, not only in terms of blending western & Indigenous culture but also in the way the SE combines social needs with business responsibilities.	Seeks to work with Indigenous employers & employees of SEs to enable them to balance both their cultural needs & the business needs of the SE.	Can be concerned about the degree to which Indigenous cultural values or practices impact on the sustainability of the social enterprise. Due to the lack of Indigenous control, Western values take precedence.	While the SE may be for Indigenous people, it does not blend Indigenous cultural values and Western values in a meaningful way. The only form of hybridity is between social and business needs.

^1^ Note while there were 63 documents that contained descriptive detail of the ownership and management of Indigenous social enterprises, some documents referred to multiple examples.

## Data Availability

Not applicable.

## Supplementary Materials

The following are available online at https://www.mdpi.com/article/10.3390/ijerph192114478/s1. Table S1: Documents in scoping review and sources. File S1: Data Extraction Tool (variables with dropdowns).

## Appendix A

**Table A1:** Mission and activities of Indigenous social enterprises (*n* = 115).

	*n*	%
**Mission**		
Community Development	36	31
Employment/poverty alleviation	33	29
Health specific	2	2
Rehabilitation	1	1
Well-being	2	2
Education/training	1	1
Not stated or unable to determine	40	35
**Activity of SE**		
Agriculture, forestry and fishing	7	6
Arts and recreation services	5	4
Computer system design and related services	2	2
Construction	1	1
Education and training	4	3
Electricity, gas, water and waste services	1	1
Health care and social assistance	4	3
Hospitality—accommodation and food service	8	7
Mining	1	1
Tourism	8	7
Trade—wholesale and retail	1	1
Multiple activities (more than one SE or SE with multiple activities)	52	45
Not stated or unable to determine	21	18

**Table A2:** Relationship between mission and activities of Indigenous social enterprises.

Mission of SE	Activities
Community Development	Construction (*n* = 1) Electricity, gas, water and waste services (*n* = 1) Health care and social assistance (*n* = 1) Hospitality—accommodation and food service (*n* = 3) Tourism (*n* = 6) Multiple activities /unable to determine (*n* = 18)
Employment/poverty alleviation	Agriculture, forestry and fishing (*n* = 5) Arts and recreation services (*n* = 5) Computer system design and related services (*n* = 1) Education and training (*n* = 2) Hospitality—accommodation and food service (*n* = 3) Mining (*n* = 1) Tourism (*n* = 2) Trade—wholesale and retail (*n* = 1) Multiple activities/unable to determine (*n* = 13)
Health specific	Health care and social assistance (*n* = 2)
Rehabilitation	Education and training (*n* = 1)
Wellbeing	Health care and social assistance (*n* = 1) Computer system design and related services (*n* = 1)
