# Supplementary material for: Indigenous Social Enterprises and Health and Wellbeing: A Scoping Review and Conceptual Framework
Source: Int J Environ Res Public Health. 2022 Nov 4;19(21):14478. doi: 10.3390/ijerph192114478 (PMC9657732; doi:10.3390/ijerph192114478)
Supplement: Supplementary file 1 [file ijerph-19-14478-s001.zip › Table S1 - Documents in scoping review and sources.pdf]

Document details	Reference number	Source
Aimers, J.; Walker, P. Community development in Aotearoa New Zealand: A historical journey. Whanake: *The Pacific Journal of Community Development*, **2016**, *2*, 3-12.	98	Google
Aimers, J.; Walker, P. Can community development practice survive neoliberalism in Aotearoa New Zealand? *Community Dev. J.* **2016**, *51*, 332-349.	28	Google Scholar
Amoamo, M.; Ruckstuhl, K.; Ruwhiu, D. Balancing Indigenous Values Through Diverse Economies: A Case Study of Māori Ecotourism. *Tour. Plan. Dev.* **2018**, *15*, 478–495. https://doi.org/10.1080/21568316.2018.1481452	15	Web of Science
Anderson, R.B.; Dana, L.P.; Dana, T.E. Indigenous land rights, entrepreneurship, and economic development in Canada: "Opting-in" to the global economy. *J. World Bus.* **2006**, *41*, 45–55. https://doi.org/10.1016/j.jwb.2005.10.005.	12	Scopus
Austin, B. Success Factors for Indigenous Wildlife-Based Enterprise in Northern Australia. Ph.D. Thesis, Charles Darwin University, Darwin, Australia, 2012.	65	Google
Barraket, J.; Douglas, H.; Eversole, R.; Mason, C.; McNeill, J.; Morgan, B. Classifying social enterprise models in Australia. *Soc. Enterp. J.* **2017**, *13*, 345–361.	13	Google Scholar
Beetson, S.J.; Pradhan, S.; Gordon, G.; Ford, J. Building a Digital Entrepreneurial Platform through Local Community Activity and Digital Skills with Ngemba First Nation, Australia. *Int. Indig. Policy J.* **2020**, *11*, 1–19.	71	Web of Science
Borquist, B.; de Bruin, A. Faith-based social entrepreneurship: Towards an integrative framework. In Proceedings of the Massey University Social Innovation and Entrepreneurship Conference: Collaborating for Impact, Massey University, Auckland, New Zealand 10 - 12 February 2016	99	Google Scholar
Broad, G.; Ortiz, J. Sparking Social Transformation through Cycles of Community-Based Research. *Canadian Journal of Non-profit and Social Economy Research*. **2020,** *11*, 62–75.	100	ProQuest
Brouard, F.; McMurtry, J.; Vieta, M. Social Enterprises Models in Canada: Ontario. *Canadian journal of non-profit and social economy research,* **2015**. *6*, 63–85.	101	Google Scholar
Brueckner, M.; Paulin, S.; Davis, J.; Chatterjee, S. A case for social enterprise: At the bottom of the top of the pyramid. *Int. J. Environ. Cult. Econ. Soc. Sustain.* **2010**, *6*, 149–166.	31	Google Scholar
Brueckner, M.; Spencer, R.; Wise, G.; Marika, B. A third space social enterprise: Closing the gap through cross-cultural learning. *Aust. Aborig. Stud.* **2016**, *2,* 18–32.	17	Academic Search
Budka, P. From marginalization to self-determined participation. *J. Des Anthropol.* **2015**, *142-143* 127–153. https://doi.org/10.4000/jda.6243	48	Google Scholar
Colbourne, R. Indigenous entrepreneurship and hybrid ventures. In *Advances in Entrepreneurship, Firm Emergence and Growth*; Emerald Group Publishing Ltd.: Bentley, UK, 2017; Volume 19, pp. 93–149.	51	Scopus
Colton J.; Whitney-Squire K. Exploring the relationship between aboriginal tourism and community development. *Leisure/ Loisir*. 2010, *34*, 261–278. https://doi.org/10.1080/14927713.2010.521321	102	Scopus

Cowie, J. He Pāpori Hinonga Whakamoe: Exploring Contributions to the Indigenous Social Enterprise Network in New Zealand. Master's Thesis, Vitoria University of Wellington, Wellington, New Zealand, 2016.	61	Google Scholar
Curry, J.A.; Donker, H.; Michel, P. Social entrepreneurship and indigenous people. *J. Co-Oper. Organ. Manag.* **2016**, *4*, 108–115. https://doi.org/10.1016/j.jcom.2016.09.002.	25	Scopus
Curry, J.; Donker, H.; Krehbiel, R. Development corporations in aboriginal communities: The Canadian experience. *J. Dev. Entrep.* **2009**, *14*, 1–19. https://doi.org/10.1142/S1084946709001119.	11	Scopus
Davidson-Hunt, I.; Turner, K. Indigenous communities, the bioeconomy and natural resource development. *Journal of Enterprising Communities: People and Places in the Global Economy*. 2012, *6* https:// doi.org/10.1108/jec.2012.32906caa.001	103	Google Scholar
de Bruin, A.; Read, C. Towards understanding social innovation in multicultural societies: Implications of Māori cultural values for social innovation in New Zealand. Social Enterprise Journal. **2018,** *14,* 194–207.	104	Google Scholar
Dey, K.: Grant, S. Māori communities as social enterprise. In *Social entrepreneurship and enterprise: Concepts in context*; Tilde University Press: Melbourne, Australia, 2014; pp.194-216.	105	Google Scholar
Diamantopoulos, M.; Findlay, I. Growing pains: Social enterprise in Saskatoon's core neighbourhoods. Linking, Learning, Leveraging Social enterprises knowledgeable economies, and sustainable communities. Community-University Institute for Social Research: Saskatoon, Canada 2007	106	Google Scholar
Diaz, E; Gradilla, R. Creating a Community Development Corporation for the Brothertown Indians of Wisconsin. Harvard University Native American Program: Cambridge, MA, USA, 2003.	107	Google
Diochon, M. Social Entrepreneurship and Effectiveness in Poverty Alleviation: A Case Study of a Canadian First Nations Community. *J. Soc. Entrep.* **2013**, *4*, 302–330. https://doi.org/10.1080/19420676.2013.820779.	64	Scopus
Douglas, H. Embracing hybridity: A review of social entrepreneurship and enterprise in Australia and New Zealand. *Third Sect. Rev.* **2015**, *21*, 5–30.	18	Google Scholar
Elson, P.; Hall, P.; Leeson-Klym, S. Social enterprises in the Canadian West. *Canadian Journal of Nonprofit and Social Economy Research*. **2015**, *6,* 83–103	108	Google Scholar
Employment and Social Development Canada. *Inclusive innovation: new ideas and new partnerships for stronger communities.* Employment and Social Development Canada: Canada. 2018.	109	Google
Farrelly T. Community-based ecotourism as indigenous social entrepreneurship. In *The Routledge Handbook of Tourism and Environment*; Routledge: London, UK	110	Google Scholar
Ferguson, E.; Ferguson, M. Social Enterprise Scoping Study: Indigenous Food Needs and Opportunities in Northern Manitoba. Ph.D. Thesis, University of Calgary, Calgary, AB, Canada, 2017.	62	Google
First Peoples Group. Aboriginal Economic Development in Canada: Best Practices, Policies and Strategies. First Peoples Group. Available online: http://www.firstpeoplesgroup.com/mnsiurban/PDF/economic_development/Aboriginal_Economic_Development_In_Canada.pdf (accessed on 4 November 2022).	111	Google

Gerrard, J. Welfare rights, self-help and social enterprise: Unpicking neoliberalism's mess. *J. Sociol.* **2017**, *53*, 47–62.	29	Google Scholar
Goodfellow-Baikie, R.; English, L. First Nations and community economic development: A case study. *Community Development Journal*. **2006**, *41*, 223-33.	112	Scopus
Gorman, J.T.; Bentivoglio, M.; Brady, C.; Wurm, P.; Vemuri, S.; Sultanbawa, Y. Complexities in developing Australian Aboriginal enterprises based on natural resources. *Rangel. J.* **2020**, *42*, 113–128. https://doi.org/10.1071/RJ20010.	21	Scopus
Grant, S. Social enterprise in New Zealand: An overview. *Soc. Enterp. J.* **2017**, *13*, 410–426. https://doi.org/10.1108/sej-09-2017-0046.	16	Web of Science
Grant, S. Contextualising social enterprise in New Zealand. *Soc. Enterp J.* **2008,** *4*, 9–23.	113	Google Scholar
Grant, S. Social enterprise through a critical appreciative lens. In *Social enterprise. Accountability and evaluation around the world;* Routledge. London, UK, 2014; pp. 213-232.	114	Google Scholar
Greenwood, L.; Nash, T.; Whitehead, E. *Transforming Our Economy: Financing the Social Enterprise Sector in Aotearoa New Zealand*. Department of Internal Affairs, The Ākina Foundation and the Community Enterprise Network Trust (CENT). Wellington, New Zealand. 2018.	115	Google
Hardy, K. Yukon Coops final report for MBA (CED), Cape Breton University, Sydney, Canada, 2009.	116	Google
Harrington, C.; Clarkson, G.S. Native American Approaches to Social Entrepreneurship. In *Mission-Driven Approaches in Modern Business Education*; IGI Global: Hershey, PA, USA, 2019; pp. 46–61.	91	Google Scholar
Harris, J.; McLeod, R. Partnering to Build a Social Co-operative for Aboriginal Women Transitioning from Prison. *Journal of Co-operative Studies*, **2014**, *47*, 25-38.	117	Google Scholar
Harrison, L.; Hickey, C.; Campbell, P. *Capacity Building and Social Enterprise: Individual and Organisational Transformation in a Transitional Labour Market Program*; Deakin University: Melbourne, Australia, 2015.	58	Google
Henare, M.; Lythberg, B.; Woods, C. Teaming with intent: Harmonising heritage, innovation and multiple generations within the Māori entrepreneurial team. *Bus. Manag. Rev.* **2014**, *5*, 465–476.	77	Google Scholar
Henriques, I.; Colbourne, R.; Peredo, A.M.; Anderson, R.B. Relational and social aspects of Indigenous entrepreneurship: The hupacasath case. In *Indigenous Wellbeing and Enterprise: Self-Determination and Sustainable Economic Development*; Routledge: London, UK, 2020; pp. 313–340. https://doi.org/10.4324/9780429329029-13.	14	Scopus
Henry, E.; Dana, L.P. Māori indigenous research: Impacting social enterprise and entrepreneurship. In *A Research Agenda for Social Entrepreneurship*; Edward Elgar Publishing: Cheltenham, UK, 2019; pp. 128–135.	70	Scopus
Henry, E.; Newth, J.; Spiller, C. Emancipatory Indigenous social innovation: Shifting power through culture and technology. *J. Manag. Organ.* **2017**, *23*, 786–802. https://doi.org/10.1017/jmo.2017.64.	63	Scopus
Hernandez, G. Indigenous Perspectives on Community Economic Development: A North-South Conversation. *Can. J. Nonprofit Soc. Econ. Res.* **2013**, *4*, 6–24.	60	Academi c Search

Hotte, N., Nelson, H., Hawkins, T., Wyatt, S., & Kozak, R. Maintaining accountability between levels of governance in Indigenous economic development: Examples from British Columbia, Canada. Canadian Public Administration, **2018** *61*, 523-549. doi:10.1111/capa.12287	118	Scopus
Howard-Wagner, D. Successful urban Aboriginal-driven community development: a place-based study of Newcastle. ANU Centre for Aboriginal Economic Policy Research CAEPR: Canberra, Australia, 218.	119	Google
Indigenous and Northern Affairs Canada. Evaluation of the Urban Aboriginal Strategy. Indigenous and Northern Affairs Canada. 2017	120	Google
Islam, D.; Berkes, F. Between a business and a social enterprise, The Norway House Fisherman's Co-op, northern Manitoba, Canada. Journal of Enterprising Communities-People and Places of Global Economy, **2017**, *11*, 530-546. doi:10.1108/jec-06-2016-0018	121	Web of Science
Jackson, B.; Nicoll, M.; Roy, M. The distinctive challenges and opportunities for creating leadership within social enterprises. *Social Enterprise Journal*, **2018** *14*, 71-91.	122	Google Scholar
Jackson, S. The Development of Social Enterprise in Western Australia. Mission Australia and WACOSS Initiative. Western Australia, 2015	123	Google
Jennings, D. Community Economic Development: Understanding the New Zealand Context. In Proceedings of the Australia and New Zealand Third Sector Research Conference, Christchurch, New Zealand, 18–20 November 2014.	124	Google
Jones, J.; Seet, P.; Acker, T.; Whittle, M. Barriers to grassroots innovation: The phenomenon of social-commercial-cultural trilemmas in remote indigenous art centres. *Technological Forecasting and Social Change*. **2019,** *164*, https://doi.org/10.1016/j.techfore.2019.02.003	125	Scopus
Kain, J.; Sharkey, E.; Webb, R. Municipal government support of the social economy sector. Balta - BC Alberta Social Economy Research Alliance: British Columbia, Canada, 2010.	126	Google Scholar
Kaplan, M. Growing the Next Generation of Social Entrepreneurs and Start-ups in New Zealand. Fulbright New Zealand: Wellington, New Zealand, 2013	127	Google
Kawharu, M. Reinterpreting the value chain in an indigenous community enterprise context. *J. Enterprising Communities People Places Glob. Econ.* **2019**, *13*, 242–262. https://doi.org/10.1108/JEC-11-2018-0079.	68	Scopus
Kerins, S. *Social Enterprise as a Model for Developing Aboriginal Lands*; ANU Centre for Aboriginal Economic Policy Research CAEPR: Canberra, Australia, 2013.	72	APO
Kernot, C.; McNeil, J. *Australian Social Enterprises: Stories of Challenge*; The University of New South Wales: Sydney, Australia, 2011.	57	Google
Kidd, S.; McKenzie, K. Social entrepreneurship and services for marginalized groups. *Ethn. Inequalities Health Soc. Care* **2014**, *7*, 3–13. https://doi.org/10.1108/EIHSC-03-2013-0004.	80	Scopus
Lemelin, R.H.; Koster, R.; Youroukos, N. Tangible and intangible indicators of successful aboriginal tourism initiatives: A case study of two successful aboriginal tourism lodges in Northern Canada. *Tour. Manag.* **2015**, *47*, 318–328.	47	Google Scholar

Lewis, M. Mapping the social economy in BC and Alberta: Towards a strategic approach. BC-Alberta Social Economy Research Alliance: British Columbia, Canada, 2006.	128	Google Scholar
Ling, S. Social Enterprises and Regional Development: Opportunities and Constraints in Australia. Thesis, Queensland University of Technology, Queensland, Australia, 2013.	129	Google
Logue, D.; Pitsis, A.; Pearce, S.; Chelliah, J. Social enterprise to social value chain: Indigenous entrepreneurship transforming the native food industry in Australia. *J. Manag. Organ.* **2018**, *24*, 312–338.	50	Scopus
MacKinnon, S. The Social Economy in Manitoba. *Social Economy*. **2006**, *8*, 26.	130	Google Scholar
McCarthy, D.; Millen, M.; Boyden, M.; Alexiuk, E. et. al. A First Nations-led social innovation: a moose, a gold mining company, and a policy window. *Ecology and Society*, 2014, *19*, http://dx.doi.org/10.5751/ES-06771-190402	131	Google Scholar
McMeeking, S.; Grant, W.; Melrose, U. *Insights on Māori Social Enterprise 2017-Pakihi What Kaupapa*; University of Canterbury Research Repository: Christchurch, New Zealand, 2017.	20	Google Scholar
McMurtry, J.; Brouard, F. Social enterprises in Canada: An introduction. *Canadian journal of non-profit and social economy research*, **2015**, *6*, .	132	Google Scholar
Métis Nation of Ontario, Ontario Federation of Indigenous Friendship Centres, and Ontario Native Women's Association. The Social Innovation Research Project. Métis Nation of Ontario. 2015	133	Google
Morley, S. Success factors for Indigenous entrepreneurs and community-based enterprises. Resource sheet no. 30 produced for the Closing the Gap Clearinghouse: Canberra, Australia, 2014.	134	APO
Morrison, M.; Collins, J.; Basu, P.; Krivokapic-Skoko, B. *Determining the Factors Influencing the Success of Private and Community-Owned Indigenous Businesses Across Remote, Regional and Urban Australia*; Charles Sturt University: Bathurst, Australia, 2014	30	Other
Murphy, H. Learning on Country with Bana Yarralji Buba: Educational Tourism and Aboriginal Developmental Aspirations. Ph.D. Thesis, James Cook University, College of Marine and Environmental Sciences, Townsville, Australia, 2015.	74	Google Scholar
Murphy, H.; Wallace, M. *Engaging with the Social Economy in Aboriginal Australia: The Experience of Eastern Kuku Yalanji Social Entrepreneurs*; United Nations Research Institute for Social Development: Geneva, Switzerland, 2013.	66	Google Scholar
Murray, I. Indigenous benefits management structures as social enterprises: key challenges for economic development. *Journal of Energy & Natural Resources Law*, **2021** *39*, 137-158. https://doi.org/10.1080/02646811.2020.1824861	135	Academi c Search
Nandu-Templeton, J.; Vanderklei, M.; de Vries, H.; Love, T.; Hamilton, R. Interpreting the narratives of Māori entrepreneurs. *MAI Journal*, **2017**, *6*, 164-179.	136	Google Scholar
Neha, T.; Macfarlane, A.; Macfarlane, S.; Clarke, T.H.; Derby, M.; Torepe, T.; Duckworth, F.; Gibson, M.; Whelan, R.; Fletcher, J. Sustainable prosperity and enterprises for Māori communities in Aotearoa New Zealand: A review of the literature. *J. Enterprising Communities People Places Glob. Econ.* **2021**, *15*, 608–625. https://doi.org/10.1108/JEC-07-2020-0133.	69	Scopus

Novek, J. Urban food initiatives in Saskatoon and Winnipeg: Refashioning the social division of labour. *Prairie Forum*, **2009**, *34*, 333-356	137	Scopus
O'Sullivan, J.; Dana, T. Redefining Māori economic development. *International Journal of Social Economics*, **2008**, *35*, 364-379.	138	Google Scholar
Overall, J.; Tapsell, P.; Woods, C. Governance and indigenous social entrepreneurship: when context counts. *Social Enterprise Journal*, **2010**, *6*, 146-161. https://doi.org/10.1108/17508611011069275	139	ProQuest
Pearson, C.A.; Helms, K. Indigenous social entrepreneurship: The Gumatj clan enterprise in east Arnhem land. *J. Entrep.* **2013**, *22*, 43–70.	75	Scopus
Pinto, L.; Blue, L. Aboriginal entrepreneurship financing in Canada: Walking the fine line between self-determination and colonization. *Journal of Entrepreneurship in Emerging Economies*, **2017,** 9, 2-20.	140	Google Scholar
Richmond, C.; Elliott, S.; Matthews, R.; Elliott, B. The political ecology of health: Perceptions of environment, economy, health and well-being among 'Namgis First Nation. *Health and Place*, **2005**, *11*, 349-365. https://doi.org/10.1016/j.healthplace.2004.04.003	141	Scopus
Rodon, T. Land Claim Organizations and the Social Economy in Nunavut and Nunavik. In *Northern Communities Working Together: The Social Economy of Canada's North*, University of Toronto Press: Toronto, Canada, 2015, 97-115.	142	Google Scholar
Rony, M. Impact Investing & Aboriginal Community Economic Development: From Fishing Net to Financial Net. University of Manitoba. Winnipeg, Canada, 2016.	143	Google
Savage, C.; Leonard, J.; Grootveld, C.; Edwards, S.; Dallas-Katoa, W. *The Evaluation of Wave One Initiatives: Te Putahitanga o Te Waipounamul*; IHI Research: Christchurch, Australia, 2016.	81	Google
Savage, C.; Leonard, J.; Goldsmith, L. *The Evaluation of Wave 2 and 3 Whānau Initiatives for Te Pūtahitanga o Te Waipounamu*; IHI Research: Christchurch, Australia, 2017.	82	Google
Savage, C.; Leonard, J.; Goldsmith, L. *The Evaluation of Waves 4 & 5 Commissioning for Te Pūtahitanga o Te Waipounamu*; IHI Research: Christchurch, Australia, 2018.	83	Google
Savage, C.; Leonard, J.; Te Hemi, H.; Hynds, A.; Dallas-Katoa, W.; Goldsmith, L. *The Evaluation of Wave 6 Whānau Initiatives for Te Pūtahitanga o Te Waipounamu*; IHI Research: Christchurch, Australia, 2018.	84	Google
Scherrer, P. Tourism to serve culture: The evolution of an Aboriginal tourism business model in Australia. *Tour. Rev. AIEST—Int. Assoc. Sci. Expert. Tour.* **2020**, *75*, 663–680. https://doi.org/10.1108/TR-09-20190364	54	ProQuest
Seet, P.; Jones, J.; Acker, T.; Jogulu, U. Meaningful careers in social enterprises in remote Australia: employment decisions among Australian Indigenous art centre workers. *International Journal of Human Resource Management*. **2018,** *32,* 1643-1684**,** https://doi.org/10.1080/09585192.2018.1528556	144	Scopus
Sengupta, U.; Vieta, M.; McMurtry, J. Indigenous Communities and Social Enterprise in Canada. *Can. J. Nonprofit Soc. Econ. Res.* **2015**, *6*, 104–123.	59	Academic Search

Sengupta, U. The future of social economy leadership and organizational composition in Canada: Demand from demographics, and difference through diversity. *Rev. Interv. Économiques Pap. Political Econ.* **2016**, *54,* 2-19	79	Google Scholar
Shahidullah, A.; Islam, D. Social entrepreneurship by cooperative: Examining value chain options of an indigenous fisherman's co-op. *International Journal of Entrepreneurship and Small Business*. **2018**, *35*, 598-615. https://doi.org/10.1504/IJESB.2018.096177	145	Scopus
Simmons, D.; Bayha, W.; Fink, I.; Gordon, S.; Rice, K.; Taneton, D. 12 Gúlú Agot’ı T’á Kə Gotsúhʔa Gha (Learning about Changes): Rethinking Indigenous Social Economy in Délınę, Northwest Territories. In *Northern Communities Working Together: The Social Economy of Canada's North*. University of Toronto Press: Toronto, Canada, 2015, 253–274.	146	Other
Smiddy, L.O. Responding to Professor Janda-The US Experience: The Alaska Native Claims Settlement Act (ANCSA) Regional Corporation as a Form of Social Enterprise. *Vt. Law Rev.* **2005**, *30*, 823.	92	Google Scholar
Smith, D. The Aboriginal Governance and Management Program Aboriginal Peak Organisations of the Northern Territory (APONT). DESmith Consulting: Canberra, Australia, 2015.	147	Google
Smith, D. Evaluating Governance Effectiveness: A facilitated process with the board of Yarnteen Corporation In *Case Study Report No. 2 Indigenous Community Governance Project*; ANU Centre for Aboriginal Economic Policy Research CAEPR: Canberra, Australia, 2006.	76	Google
Department of Employment. Social Enterprise Development & Investment Funds (SEDIF) Evaluation Report. Department of Employment, Centre for Social Impact, Swinburne University of Technology and Social Policy Research Centre, University of New South Wales, Canberra, Australia, 2016. Available online: https://www.dewr.gov.au/sedif/resources/social-enterprise-development-investment-funds-sedif-evaluation-report (accessed on 4 November 2022).	148	Google
Sousa, J.; Hamdon, E.; Preliminary profile of the size and scope of the social economy in Alberta and British Columbia. Balta - BC-Alberta Social Economy Research Alliance: University of Alberta, Alberta, Canada, 2010.	149	Google Scholar
Spencer, R.; Brueckner, M.; Wise, G.; Marika, B. Capacity development and Indigenous social enterprise: The case of the Rirratjingu clan in northeast Arnhem Land. *J. Manag. Organ.* **2017**, *23*, 839–856. https://doi.org/10.1017/jmo.2017.74.	52	Scopus
Spencer, R.; Brueckner, M.; Wise, G.; Marika, B. Australian indigenous social enterprise: Measuring performance. *J. Enterprising Communities* **2016**, *10*, 397–424. https://doi.org/10.1108/JEC-10-2015-0050.	27	Scopus
Social Ventures Australia (SVA). *Indigenous Social Enterprise Fund: Lessons Learned*; Indigenous Business Australia (IBA): Sydney, Australia, 2016.	56	APO
Tapsell, P.; Woods, C. A spiral of innovation framework for social entrepreneurship: Social innovation at the generational divide in an indigenous context. *E:CO Emerg. Complex. Organ.* **2008**, *10*, 25–34.	88	Scopus
Tapsell, P.; Woods, C. Social entrepreneurship and innovation: Self-organization in an indigenous context. *Entrep. Reg. Dev.* **2010**, *22*, 535–556. https://doi.org/10.1080/08985626.2010.488403.	78	Scopus

Tedmanson, D.; Guerin, P. Enterprising social wellbeing: Social entrepreneurial and strengths-based approaches to mental health and wellbeing in "remote" Indigenous community contexts. *Australasian Psychiatry*, **2011**, *19*, S30-S33. https://doi.org10.3109/10398562.2011.583078	150	Scopus
Thompson, J. *Evaluation of Tjanpi Desert Weavers Employment and Income Generation' Project*; Caritas Australia: *'Indigenous* Sydney, Australia, 2014.	55	Google
Vining, A; Richards, J. Indigenous economic development in Canada: Confronting principal-agent and principal-principal problems to reduce resource rent dissipation. *Resources Policy*, **2016**, *49*, 358-367. doi:10.1016/j.resourpol.2016.07.006	151	Web of Science
Volynets, I. Social innovation and Aboriginal communities. UAKN National Secretariat, National Association of Friendship Centres. McGill University, Institute for the Study of International Development, Montreal, Canada, 2015.	152	Google Scholar
Wallace, R.; Manado, M.; Agar, R.; Curry, C. Working from our strengths: Indigenous community engagement through enterprise development and training. Learning Communities: *International Journal of Learning in Social Contexts*. **2009**, December, 104-122.	153	Google Scholar
Wanasuk, P.; Thornton, T.F. Aboriginal Tourism as Sustainable Social-Environmental Enterprise (SSEE): A Tlingit Case Study from Southeast Alaska. *Int. Indig. Policy J.* **2015**, *6*, 8.	94	Google Scholar
Williams, D. First Nations Technology Council Three Year Social Enterprise Business Plan, Simon Fraser University, Vancouver, Canada, 2015.	154	Google
Wilson, G.N.; Alcantara, C. Mixing politics and business in the Canadian arctic: Inuit corporate governance in nunavik and the inuvialuit settlement region. *Can. J. Political Sci.* **2012**, *45*, 781–804. https://doi.org/10.1017/S0008423912000996.	90	Scopus
Wood, D.; Loney, S.; Taylor, K. Social enterprise and the solutions economy: A toolkit for Manitoba First Nations. AKI Energy: Winnipeg, Manitoba, Canada, 2015.	155	Google Scholar
Wuttunee, W. *Living Rhythms: Lessons in Aboriginal Economic Resilience and Vision*. McGill-Queen's University Press: Montreal, Canada, 2004.	156	Scopus
Zhang, D.; Swanson, L, Linking social entrepreneurship and sustainability. *Journal of Social Entrepreneurship*, **2014**, *5*, 175-191.	157	Google Scholar
